# The global regulatory landscape of stem cell medical aesthetics: challenges, comparisons, and pathways to coordination

**DOI:** 10.1093/stcltm/szaf079

**Published:** 2026-03-26

**Authors:** Yiding Xiao, Zirui Liao, Hailin Zhang, Tianjia Guan

**Affiliations:** Department of Plastic and Aesthetic Surgery, Peking Union Medical College Hospital, Chinese Academy of Medical Sciences, Beijing, People’s Republic of China; National Clinical Research Center for Infectious Disease, Shenzhen Third People’s Hospital, Shenzhen, People’s Republic of China; Department of Plastic and Aesthetic Surgery, Peking Union Medical College Hospital, Chinese Academy of Medical Sciences, Beijing, People’s Republic of China; School of Health Policy and Management, Chinese Academy of Medical Sciences & Peking Union Medical College, Beijing, People’s Republic of China

**Keywords:** Stem cells, medical aesthetics, regulatory framework, global governance, risk classification, cell therapy, regenerative medicine, regulatory coordination

## Abstract

This paper systematically examines the regulatory frameworks of major regulatory bodies—including the United States, the European Union, Japan, South Korea, and China—regarding the use of stem cells and their derivatives (such as conditioned media and exosomes) for cosmetic purposes. Despite the rapid global growth of “stem cell medical aesthetics” within the cosmetic medical market, its scientific evidence remains insufficient, and it faces the core challenge of inconsistent regulatory systems. Through in-depth comparative analysis, this paper not only reveals profound differences among nations in risk perception, regulatory philosophy, and terminology definitions, but also dissects the resulting market fragmentation and patient safety risks. Based on this, the paper proposes feasible pathways to advance international regulatory coordination: The primary task is to unify core definitions and classification standards (especially for derivatives like exosomes) based on scientific consensus, and establish a risk-based tiered regulatory mechanism that clearly distinguishes medical from cosmetic applications. Concurrently, fundamental research should be strengthened and specific efficacy assessment standards (e.g., instrument measurements and patient-reported outcomes) should be developed. Crucially, this paper advocates for a hybrid regulatory model integrating conditional approval with real-world evidence tracking as a pragmatic solution balancing patient safety and technological innovation. This research aims to provide theoretical support and practical guidance for constructing a global governance framework that safeguards safety while accommodating innovation efficiency.

## Graphical abstract

**Graphical Abstract:**
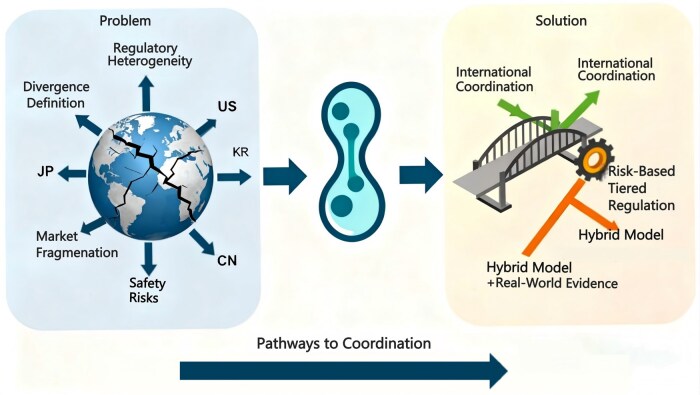

Significance Statement The global challenge of “scientific claims” coexisting with a “regulatory vacuum” in the application of stem cell technologies within medical aesthetics highlights a gap in current literature regarding systematic deconstruction of the root causes underlying regulatory divergence. Through in-depth paradigm comparisons of the regulatory frameworks in the United States, the European Union (EU), Japan, South Korea, and China, this study attributes, for the first time, the root of regulatory heterogeneity to a tripartite structural divide in risk perception, regulatory philosophy, and terminology definition. It further evaluates the chain of effects stemming from this divide—ranging from the creation of technological barriers to market disorder. Building on this analysis, the paper proposes a coordination pathway centered on unifying core international definitions, implementing risk-stratified management, and constructing hybrid regulatory models. This framework provides a actionable roadmap for policymakers, regulatory agencies, and industry stakeholders to address current challenges and strategize for future development.

## Introduction

According to Global Market Insights, the global medical aesthetics market attained a valuation of USD 19.2 billion in 2023, with a projected compound annual growth rate (CAGR) of 12% from 2024 to 2032.^1^ Concurrently, applications utilizing stem cells and their derivatives—such as conditioned media and exosomes—for aesthetic purposes have garnered substantial interest from both industry and academia in recent years. Market projections indicate that the global stem cell market will surpass USD 31 billion by 2030, with the cosmetic and anti-aging segment recognized as one of the most rapidly expanding application domains.^2^

Marketing for stem cells and their derivatives often highlights significant potential in skin regeneration, rejuvenation, and anti-aging. However, this emerging field faces severe challenges regarding scientific rigor and regulatory alignment. Technological breakthroughs are driving application diversification: mesenchymal stromal cell therapies have gained approval, induced pluripotent stem cells are evolving toward universal treatments, and exosome-based drug delivery systems enable targeted repair at cellular boundaries. These technologies and products now span aesthetic scenarios including anti-aging and skin regeneration. Yet, within the commercial interventions currently labeled “stem cell medical aesthetics,” numerous product efficacy claims lack support from randomized controlled trials. Furthermore, the absence of legally binding, globally unified regulatory definitions^3^ has resulted in a regulatory vacuum coexisting with conceptual misuse.

Against this backdrop, this paper aims to conduct an in-depth review of regulatory paradigms for stem cell medical aesthetics applications across major regulatory bodies—including the United States (US), the European Union (EU), Japan, South Korea, and China. It seeks to deconstruct the institutional roots of regulatory heterogeneity and, based on this analysis, explore feasible mechanisms for achieving international regulatory coordination. This effort provides theoretical support for constructing a global governance framework that balances safety assurance with innovation efficiency.

## Conceptual definition and scope of analysis

It must be clarified that “stem cell medical aesthetics” is not an internationally recognized scientific or regulatory classification. Rather, it serves as a collective term for a range of clinical practices and commercial activities involving the application of stem cells and their bioactive components for cosmetic intervention purposes. Although numerous academic papers and reviews have systematically outlined the potential roles and clinical advancements of stem cells in aesthetic medicine (e.g. skin repair, scar improvement, anti-aging),^4^^,^^5^ none of the major existing regulatory systems such as the U.S. Food and Drug Administration (FDA), the European Medicines Agency (EMA), or China’s National Medical Products Administration (NMPA), formally classify “stem cell medical aesthetics” as a drug or medical device. The term does not appear in their official guidelines. While relevant literature frequently discusses stem cell applications in cosmetic medicine, it continues to categorize them under “stem cell therapy”^6^ or “regenerative medicine”^7^ rather than as an independent classification. This paper adopts the term to focus on how global regulatory systems address this interdisciplinary field merging biotechnology, medical aesthetics, and consumer healthcare.

To define the scope of this review, applications were distinguished as “medical” or “cosmetic,” following established regulatory and clinical frameworks. Specifically, medical applications aim to treat or mitigate a pathological condition—such as radiation-induced skin damage or scar revision—and are generally defined by intent to restore, correct, or modify physiological function in response to disease, trauma, or congenital abnormality In contrast, cosmetic applications are designed to enhance appearance in otherwise healthy individuals, such as through anti-aging interventions or facial rejuvenation, without addressing underlying pathology.^8^ Our analysis focuses on the contentious domain wherein this boundary becomes obscured—such as in post-acne scarring, alopecia, or certain forms of tissue regeneration—where interventions may straddle therapeutic benefit and aesthetic enhancement.^9^^,^^10^ Technologies purely intended for disease treatment, with no aesthetic component or ambiguity, were excluded from this review.

The core analytical scope of this paper includes:

1. *Cell therapy applications* **:** Covering minimally processed autologous cells and in vitro expanded cells used in aesthetic indications such as facial rejuvenation and hair regeneration. For example, autologous adipose-derived stem cells (ADSCs) combined with autologous fat grafting (CAL) significantly enhance facial skin elasticity, thickness, and overall satisfaction.;^11^ the multi-level regulatory role of bone marrow/adipose-derived mesenchymal stromal cells (MSCs) injected into the scalp in skin and hair follicle regeneration, particularly through immune modulation and paracrine factors that promote hair follicle stem cell activity.^12^
2. *Derivative Product Applications* **:** Functional skincare products and injectable aesthetic products utilizing non-cellular components—such as stem cell secretome (e.g., conditioned medium, CM), exosomes, cytokines, etc—as active ingredients.^13^Notably, the International Society for Cell & Gene Tissue Therapy (ISCT) has recommended replacing the traditional term “mesenchymal stem cells” with “mesenchymal stromal cells” (MSCs). This revision aims to more accurately reflect that the therapeutic mechanisms of these cells primarily stem from their immunomodulatory and paracrine functions, rather than the previously emphasized multipotent differentiation potential.^14^Regulatory ambiguity is particularly acute for exosome-based products. As highlighted in a recent comprehensive analysis,^15^ exosomes straddle multiple regulatory categories: classified as cosmetics in South Korea if topically applied, as biologics in the U.S. if injected, and as ATMPs in the EU if derived from cultured cells. This inconsistency underscores the urgent need for global harmonization. We incorporate insights from this study to refine our proposed classification framework.

## Comparative analysis of global regulatory frameworks

### United States

The U.S. FDA classifies human cell and tissue products (HCT/Ps) under two regulatory categories based on Section 361 of the Public Health Service Act (PHS Act) and Section 351 of the Federal Food, Drug, and Cosmetic Act (FD&C Act):^16^

Low-Risk Pathway (Section 361): Must satisfy all four criteria—(1) Allogeneic use; (2) Minimal manipulation; (3) No systemic combination with exogenous components; (4) No systemic metabolic effects. Products under this pathway require only facility registration and product listing, exempting them from premarket approval. Typical applications include SVF in autologous fat grafting; 147 such products were registered with the FDA in 2021.

High-Risk Pathway (Section 351): Products failing any criterion are classified as biological products, requiring full regulatory oversight through Investigational New Drug (IND) and Biologics License Application (BLA) processes. This includes preclinical safety evaluations, Phase III clinical trials, and GMP manufacturing inspections.

To advance stem cell therapies, the U.S. FDA enacted the 21st Century Cures Act^17^ in 2016, introducing accelerated approval pathways for Regenerative Medicine Advanced Therapies (RMAT). Regenerative medicine therapies treating life-threatening diseases may qualify for RMAT designation if preliminary clinical evidence suggests they may address unmet clinical needs, thereby accelerating approval and shortening overall product development timelines.^18^ These products include cell therapy products, therapeutic tissue engineering products, human cell and tissue products, and certain gene therapies.

This regulatory framework is clearly illustrated by the clinical use of adipose-derived stem cells (ADSCs).^19^ The stromal vascular fraction (SVF), obtained from fat tissue through minimal manipulation like mechanical emulsification and centrifugation for same-session, autologous grafting, typically falls under the Section 361 pathway. In contrast, ADSC products that undergo more than minimal manipulation, such as laboratory culture expansion and incubation, are classified as biological drugs under Section 351, requiring rigorous IND and BLA approval due to their altered characteristics and potential systemic risks, such as promoting neoangiogenesis in oncologic patients.

### European Union

The European Union implements centralized management for gene therapy, somatic cell therapy, and tissue engineering products through the Regulation (EC) No 1394/2007 on Advanced Therapy Medicinal Products (ATMP). Stem cell products undergoing substantial manipulation—such as in vitro expansion or genetic modification—are typically classified as ATMPs. These require approval through the European Medicines Agency’s (EMA) single authorization procedure, adhering to efficacy evidence standards as stringent as those for traditional medicines.^20^

A key feature of this system is the Hospital Exemption Clause (Article 28):^21^ Member States may authorize healthcare institutions to use ATMPs without marketing authorization under specific conditions, including (1) individualized customization; (2) non-industrial scale production; (3) compliance with quality standards; and (4) addressing specific unmet medical needs.

However, the EMA and national medicines regulatory authorities, such as the UK’s Medicines and Healthcare products Regulatory Agency (MHRA), Germany’s Federal Institute for Drugs and Medical Devices (BfArM) have repeatedly issued warnings emphasizing that stem cell therapies used for cosmetic purposes without formal ATMP approval are illegal. They caution consumers that such treatments carry significant safety risks and lack reliable scientific evidence supporting their claimed effects.^22^ A notable case is the unauthorized use of adipose-derived MSCs in private clinics across Germany and Spain under the Hospital Exemption, despite EMA warnings.^23^

### Japan

Japan has established a unique dual-track system combining “medical technology approval + drug marketing authorization”: The 2014 Act on the Safety of Regenerative Medicine regulates regenerative projects as medical technologies, categorizing them into Classes I-III based on risk. High-risk Class III projects require special approval from the Ministry of Health, Labour and Welfare (MHLW).^24^ Meanwhile, the Pharmaceutical and Medical Devices Act regulates industrially produced cell products, which must undergo full review by the Pharmaceuticals and Medical Devices Agency (PMDA).

Act on the Safety of Regenerative Medicine: Classifies regenerative medical technologies into risk categories I-III. Category I (high risk) requires special approval from the Ministry of Health, Labour and Welfare, while Category III (low risk) undergoes review by institutional ethics committees. Approval cycles are shortened to 3–12 months, significantly accelerating clinical translation. Pharmaceuticals and Medical Devices Act: Regulates regenerative medicine products classified as drugs, requiring comprehensive review by the Pharmaceuticals and Medical Devices Agency (PMDA) with full safety and efficacy data.^25^

Notably, Japan has never approved any cell or gene therapy products primarily intended for “cosmetic” purposes through formal drug approval channels. The vast majority of so-called “stem cell beauty” treatments marketed in Japan exploit provisions in the Act on Safety Assurance of Regenerative Medicine for low-risk autologous cell processing, offered as out-of-pocket medical services within healthcare facilities. These treatments lack scientific validation and are not considered approved “drugs” or “products,” but rather “medical procedures.”

### South Korea

The Ministry of Food and Drug Safety (MFDS) of South Korea regulates cosmetics in accordance with internationally accepted principles. The Regulations on Cosmetic Safety Standards, etc explicitly prohibit the inclusion of live human cells, tissues, or their inadequately purified derivatives as cosmetic ingredients.^26^ High-risk products (e.g., gene-edited stem cells) require additional ethical review and special permits, while low-to-medium risk products (e.g., autologous adult stem cells) may undergo simplified approval processes but must still meet GMP/GLP standards.^27^ Due to their potential biosafety risks (e.g., immunogenicity, infection, or tumorigenicity), such substances are classified as biopharmaceuticals or regenerative medicine products. They must undergo rigorous preclinical and clinical trials^28^ and obtain MFDS marketing authorization as “cell therapy products” or “biological products” before being used for medical purposes.^29^

Despite Korea’s active advancement in regenerative medicine—such as promoting conditional approval for stem cell-based innovative therapies through the “Guidelines for Cell and Gene Therapy Products” (e.g., the cartilage repair product Cartistem)—these products fall under the category of prescription medical devices, fundamentally distinct from cosmetics.^30^Furthermore, South Korea’s revised “Act on the Safety and Support of Advanced Regenerative Medicine and Biopharmaceuticals,” enacted in February 2025, stipulates that patients suffering from “serious, rare, or incurable” diseases who are not participating in clinical trials may receive stem cell treatments still in the research phase but with proven safety and efficacy. Medical institutions or companies may charge fees to support treatment implementation. However, South Korean law explicitly prohibits the use of stem cells for cosmetic anti-aging purposes.

### China

Mainland China has uniformly incorporated stem cell-based medical aesthetics products into its pharmaceutical regulatory framework. According to the revised Drug Administration Law (2019) and the Technical Guidance Principles for Research and Evaluation of Cell Therapy Products (2017), any cell therapy involving in vitro expansion, genetic modification, or allogeneic sources must complete the full drug approval process (IND/BLA).^31^ In contrast, Hainan’s Boao Lecheng International Medical Tourism Pilot Zone adopts a “dual-track system + dynamic risk control” model. Leveraging national policy authorizations from 2013 and 2019,^32^ it establishes a clear pathway for stem cell clinical research. The 2024 Regulations on Promoting Biomedical New Technologies^33^ breakthrough by permitting provincial-level direct authorization for clinical applications. In 2025, two batches of technology catalogs (totaling 31 items) were released, compressing the approval cycle from 3 years to 3 days. Notably, cell therapy or exosome therapy technologies promoting skin regeneration were included in the second batch of the technology catalog.^34^

The differences among countries can be seen in Table 1.

**Table 1.** Global landscape of regulatory policies for stem cell applications in aesthetic medicine.

Country/Region	Core Regulatory Body	Key Regulations/Pathways	Regulatory Classification Method	Standard Drug Pathway (IND → Market Approval)	Special/Accelerated Pathway (if applicable)	Note
**United States**	Food and Drug Administration (FDA)	Public Health Service (PHS) Act Sections 351 and 361, in conjunction with the Federal Food, Drug, and Cosmetic (FD&C) Act	Two-Tier Pathway: Based on criteria such as “minimal manipulation” and “homologous use,” products are classified as either low-risk (regulated under Section 361) or high-risk (requiring IND/BLA approval under Section 351).	96–120 months (8–10 years)	RMAT pathway: 48–72 months (4–6 years)	Evidence-Driven; clear distinction between regulated drugs and minimally manipulated tissues; includes the Regenerative Medicine Advanced Therapy (RMAT) designation for accelerated approval.
**European Union**	European Medicines Agency (EMA)	Regulation (EC) No 1394/2007 on Advanced Therapy Medicinal Products (ATMPs)	Centralized Authorization: Stem cell products undergoing “more than minimal manipulation” or non-homologous use are classified as ATMPs and require centralized marketing authorization.	84–108 months (7–9 years)	Hospital Exemption: Immediate use (limited to individualized, non-commercial settings)	Evidence-Driven; rigorous centralized evaluation; includes a “Hospital Exemption” (Article 28) allowing use in authorized hospitals under specific conditions without full marketing authorization.
**Japan**	Ministry of Health, Labour and Welfare (MHLW)	Act on the Safety of Regenerative Medicine; Pharmaceuticals and Medical Devices Act (PMD Act)	Dual-Track System: Regenerative “medical techniques” are approved under risk-based categories (I–III) via institutional review; “products” (e.g., cell-based drugs) require full PMDA review under the PMD Act.	60–84 months (5–7 years)	Regenerative Medicine Technology pathway: 3–12 months (use restricted to healthcare institutions)	Translation-Promoting; decouples approval of clinical techniques from product development, enabling rapid translation; no approved cell therapies for purely cosmetic indications.
**South Korea**	Ministry of Food and Drug Safety (MFDS)	Cosmetics Act and its Enforcement Decree	Purpose-Oriented: The Cosmetics Act explicitly prohibits the use of live human cells in cosmetics; stem cell-based products are regulated as biologics or cell therapy products under the Pharmaceutical Affairs Act.	72–96 months (6–8 years)	Conditional Approval: 24–36 months (e.g., Cartistem)	Translation-Promoting; offers conditional-approval pathways for regenerative medicines; explicitly prohibits the use of stem cells for anti-aging or cosmetic purposes in cosmetics.
**China**	National Medical Products Administration (NMPA)	Drug Administration Law of the People’s Republic of China; Technical Guidelines for Cell Therapy Products	Unified Framework: Cell therapy products are generally regulated as biological drugs requiring IND/BLA-like pathways.	84–120 months (7–10 years)	Boao Lecheng Special Access: 3 days—3 months (limited to designated institutions within the zone)	Dual-Track in Practice: Mainland China follows strict drug regulations; the Boao Lecheng International Medical Tourism Pilot Zone implements a special access mechanism allowing use of unapproved but internationally marketed technologies under supervision, accelerating clinical adoption.

## In-depth analysis of the roots and impacts of regulatory heterogeneity

### institutional roots: the triple divide of perception, philosophy, and terminology

1. Differences in Risk Perception: The U.S. FDA emphasizes the potential theoretical risks of cell therapy, with regulatory thresholds determined by whether products meet standards such as “minimal manipulation” and “intended for use in the same species.” Products meeting these standards require only facility registration and product listing (following 21 CFR Part 1271), without formal premarket approval (e.g., IND or BLA), representing a relatively low entry barrier. Products failing to meet these criteria are classified as drugs, biological products, or devices, mandating strict premarket approval processes.^35^ This regulatory logic prioritizes objective assessments of product characteristics over theoretical risk alone. In contrast, Asian regulators like Japan’s Ministry of Health, Labour and Welfare (MHLW) and South Korea’s Ministry of Food and Drug Safety (MFDS) focus more on evaluating risk levels in practical applications—such as the low incidence of adverse events in localized autologous stem cell therapies—thereby supporting a more flexible regulatory approach.
2. Regulatory Philosophy Spectrum: The U.S. and Europe adhere to an “evidence-driven” regulatory model, requiring rigorous clinical trials to confirm safety and efficacy before cell therapy products enter the market, prioritizing patient protection. Japan and South Korea lean toward “translation-promoting” regulation, using risk-based classification systems to categorize technologies and accelerate the clinical adoption of innovative therapies. This divergence forms a global spectrum of regulatory values, with one end emphasizing evidence accumulation and the other prioritizing translation efficiency, reflecting different nations’ strategic choices in balancing innovation and safety.
3. Terminology conflicts: Significant divergences exist in operational definitions of core concepts such as “minimal manipulation” (e.g., whether fat centrifugation substantially alters tissue biological properties) and “autologous use” (whether facial injection of autologous fat complies with the principle of homogeneity, i.e., consistency between treatment site and source tissue). The U.S. may classify fat centrifugation as a manipulative procedure under drug regulations, while Japan and South Korea may treat it as minimal processing under medical device regulations. These definitional differences result in identical technologies being categorized under entirely distinct regulatory frameworks across nations, complicating international harmonization.

### Systemic impact: from technical barriers to market disorder

The evolution of cosmetic stem cell products from technical barriers to market disorder stems from the fragmentation and enforcement gaps within the global regulatory framework. Despite the high technical barriers and substantial R&D costs in this field, profound differences among nations in risk perception, regulatory philosophy, and terminology definitions compel companies to duplicate investments to meet diverse market requirements. This results in a 30%-50% surge in R&D costs,^36^ creating a “fragmented” predicament. Simultaneously, the coexistence of the U.S. and Europe’s “evidence-driven” stringent approval processes and Japan and South Korea’s “translation-promoting” flexible pathways has fostered regulatory arbitrage within policy gaps. The implementation of “Right to Try” laws in some U.S. states^37^ has created a direct conflict with federal FDA oversight, a loophole exploited by numerous clinics to market unproven and often dangerous interventions directly to consumers.^38^^,^^39^, Similarly, the EU’s Hospital Exemption, designed for unmet medical needs, is sometimes misapplied to justify commercial cosmetic cell therapies. The consequences are not merely theoretical; they manifest as severe patient harm, including cases of blindness from intravascular facial injections, severe infections, and the development of tumors from poorly characterized cell preparations.^40^^,^^41^ Instead of transforming technological barriers into regulatory ones, the institutional divide has evolved into market disorder, gravely threatening public health and hindering the industry’s sustainable development.

However, some regulatory jurisdictions have recently begun experimenting with hybrid models that integrate evidence-based and real-world approaches. For instance, Japan’s revised Pharmaceutical and Medical Devices Act introduced a “conditional approval with time limit” system, permitting innovative therapies with preliminary efficacy confirmation to enter the market while allowing real-world evidence to supplement and refine clinical data. The EU Medical Device Regulation, meanwhile, mandates “post-market follow-up studies,”embedding real-world data collection within the regulatory closed-loop system. This model establishes a dynamic assessment mechanism that avoids unduly delaying access to innovative therapies due to insufficient evidence while ensuring patient safety through continuous monitoring. Its core lies in creating a risk-proportional regulatory pathway: adopting a progressive evidence generation strategy for areas with high disease burden and unmet needs, coupled with mandatory risk management systems (such as the EU’s PRAC system) to achieve full-cycle oversight.

## Regulatory coordination pathways: building global solutions

### Establish core definitions and classification standards based on international scientific consensus

The root cause of current regulatory heterogeneity lies in the lack of unified definitions for core concepts. The World Health Organization Expert Committee on Biological Safety (ECBS) has released the document “WHO Guidance on the Development of a Global Regulatory Framework for Cell and Gene Therapy Products,” outlining regulatory approaches for cell/gene therapy products to promote harmonization and enhancement of regulatory systems among member states.^42^The 2024 World Health Assembly (WHA77.4) adopted a resolution formally placing “the safe and ethical use of human cells, tissues, and organs” on its agenda, further reinforcing WHO’s leadership role in this field.^43^

It is recommended that the WHO or the International Society for Stem Cell Research (ISSCR) lead efforts^44^ to develop a globally applicable classification and definition framework for stem cell-based medical aesthetic products. Key priorities include: - Objective criteria for “minimal manipulation” and “autologous use”: For instance, whether centrifugation of adipose tissue constitutes “minimal manipulation,” and whether facial injections of fat-derived cells comply with the autologous principle, require unified thresholds based on scientific data. Additionally, the classification of stem cell derivatives (e.g., exosomes, conditioned media) must be clarified—whether they fall under cosmetic ingredients, biological products, or medical devices—to resolve regulatory conflicts where South Korea and China classify them as cosmetics while Europe and the US regulate them as pharmaceuticals.

### Strengthen risk-based tiered regulation, distinguishing medical from cosmetic applications

Recommend implementing differentiated regulation based on application purpose (disease treatment vs. cosmetic enhancement) and risk level: - Medical-grade applications (e.g., scar repair, hair follicle regeneration) should continue under pharmaceutical regulation, requiring completion of Phase III clinical trials. However, an “adaptive approval” mechanism could be introduced—similar to China’s Hainan “pilot first” policy—granting conditional approval for products with early positive safety data.^45^ For cosmetic-grade applications, such as anti-aging skincare products, reference can be made to South Korea’s regulatory approach for stem cell culture supernatants. These should be managed as novel cosmetic ingredients, requiring local skin safety data while exempting systemic toxicity testing to accelerate market entry.

### Support basic research and translational innovation while clarifying efficacy evidence standards

Research indicates that very few stem cell-based aesthetic medicine RCTs strictly adhere to CONSORT standards, and existing RCTs predominantly focus on surgical assistance techniques rather than stem cells themselves.^46^ To address the challenge of “lack of RCT support for efficacy claims” in stem cell beauty products,^47^ establish a multinational joint research fund prioritizing basic studies like the role of stem cell paracrine mechanisms in skin regeneration to provide theoretical foundations for product efficacy. Simultaneously, establish cosmetic efficacy evaluation standards. The International Organization for Standardization (ISO) should publish guidelines for evaluating the efficacy of stem cell-based cosmetic products, standardizing testing methods for indicators like skin elasticity and wrinkle reduction. For instance, these include: (1) Instrumental measurements: instrumental-based skin elasticity measurements (specific biomechanical parameters), stratum corneum hydration assessment, and 3D optical imaging for wrinkle depth quantification and so on; (2) Validated patient-reported outcomes: such as the FACE-Q Aesthetic Module or Skindex-16 for quality-of-life impact assessment; and (3) Minimum trial duration: no less than 12 weeks to ensure the recording of biologically significant effects.(4) And other specific indicators.

### Exploring hybrid regulatory models as a pragmatic solution to harmonization barriers

Despite shared objectives, global regulatory coordination faces persistent structural barriers: disparities in healthcare financing models between Asia’s self-funded aesthetic medicine market and Europe’s insurance-dominated systems; national sovereignty concerns in biomedical innovation; and the ongoing lack of international mutual recognition for clinical data. To overcome these bottlenecks, we propose establishing a joint WHO-International Society for Stem Cell Research (ISSCR) Working Group on Regenerative Aesthetics. This group would be tasked with developing model regulations for low-risk aesthetic cell-derived products, establishing a unified adverse event registry, and piloting mutual recognition of GMP inspections across participating jurisdictions.

Against this backdrop, a hybrid regulatory model integrating “conditional approval” with “real-world evidence continuous monitoring” offers a pragmatic pathway to resolve the impasse. This dual-pathway framework achieves a dynamic equilibrium between safety and innovation through two mechanisms: First, drawing from practices like Japan’s “pioneer review” or China’s Hainan Free Trade Zone, it grants conditional marketing authorization for innovative technologies with preliminary safety and efficacy validation, significantly accelerating patient access. Second, it mandates a real-world evidence collection system requiring applicants to submit clinical efficacy data via standardized electronic health records within 24 months post-market launch. This design stimulates innovation through early access while building safety defenses through continuous monitoring. Should companies fail to submit real-world evidence on schedule, or if evidence indicates an unbalanced risk-benefit ratio, an automatic product withdrawal mechanism is triggered, thereby forming a closed-loop regulatory system.

## Limitations

This study has several limitations. First, our analysis relies primarily on publicly available regulatory documents and peer-reviewed literature, potentially missing unpublished enforcement actions or gray-market practices. Second, we focused on five major jurisdictions; while regulatory approaches in emerging markets (e.g., Brazil, India, UAE) may differ significantly. Third, our policy recommendations assume political will for international cooperation, which may be hindered by geopolitical tensions or economic competition in the regenerative medicine sector. Finally, while we cite latest research on exosome regulation,^12^ rapid technological evolution may outpace even the most agile regulatory frameworks. Future research should employ mixed methods—including stakeholder interviews and market surveillance—to capture real-world implementation gaps.

## Conclusion

This paper compares the regulatory frameworks of the the United States (US),the European Union (EU),Japan, South Korea, and China revealing that global regulatory heterogeneity stems from deep-seated differences in risk perception philosophies, weighting of evidence standards, and terminology systems. This fragmented landscape poses multiple challenges, including dispersed R&D resources and soaring compliance costs, which constrain technological innovation and jeopardize public safety. The key to overcoming this dilemma lies in promoting science-based global regulatory coordination. Relevant practices from South Korea, Japan, and China’s Hainan Province offer valuable insights for reconstructing regulatory paradigms. Future efforts should seek consensus across three dimensions: unifying core definitions, achieving mutual recognition of international standards, and adapting risk classification systems. This will establish a governance framework that balances safety and innovation.
