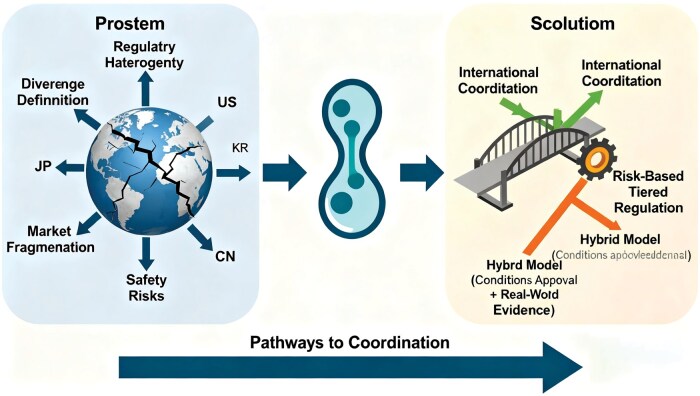# Correction to: The global regulatory landscape of stem cell medical aesthetics: challenges, comparisons, and pathways to coordination

**DOI:** 10.1093/stcltm/szag066

**Published:** 2026-08-04

This is a correction to: Yiding Xiao, Zirui Liao, Hailin Zhang, Tianjia Guan, The global regulatory landscape of stem cell medical aesthetics: challenges, comparisons, and pathways to coordination, *Stem Cells Translational Medicine*, Volume 15, Issue 4, April 2026, szaf079, https://doi.org/10.1093/stcltm/szaf079

There were errors in the originally published Graphical Abstract. These are now corrected in the article to read:

**Figure:**
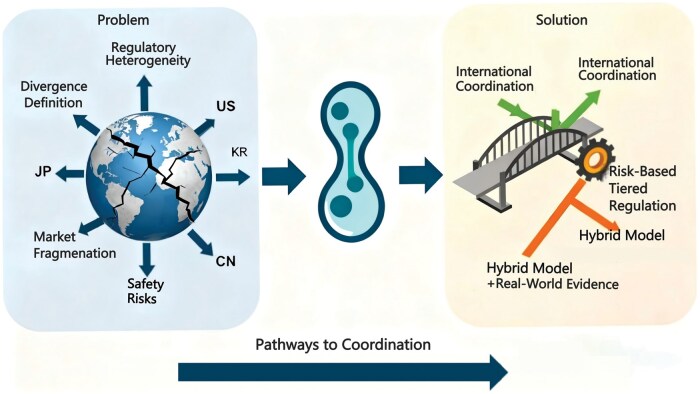
instead of:

**Figure:**